# Identification of molecular subtypes of ischaemic stroke based on immune‐related genes and weighted co‐expression network analysis

**DOI:** 10.1049/syb2.12059

**Published:** 2023-02-18

**Authors:** Duncan Wei, Xiaopu Chen, Jing Xu, Wenzhen He

**Affiliations:** ^1^ Department of Pharmacy First Affiliated Hospital of Shantou University Medical College Shantou Guangdong China; ^2^ Department of Neurology First Affiliated Hospital of Shantou University Medical College Shantou Guangdong China

**Keywords:** bioinformatics, biology, genetics, genomics

## Abstract

Immune system has been reported to play a key role in the development of ischaemic stroke (IS). Nevertheless, its exact immune‐related mechanism has not yet been fully revealed. Gene expression data of IS and healthy control samples was downloaded from Gene Expression Omnibus database and differentially expressed genes (DEGs) was obtained. Immune‐related genes (IRGs) data was downloaded from the ImmPort database. The molecular subtypes of IS were identified based on IRGs and weighted co‐expression network analysis (WGCNA). 827 DEGs and 1142 IRGs were obtained in IS. Based on 1142 IRGs, 128 IS samples were clustered into two molecular subtypes: clusterA and clusterB. Based on the WGCNA, the authors found that the blue module had the highest correlation with IS. In the blue module, 90 genes were screened as candidate genes. The top 55 genes were selected as the central nodes according to gene degree in protein–protein interactions network of all genes in blue module. Through taking overlap, nine real hub genes were obtained that might distinguish between clusterA subtype and clusterB subtype of IS. The real hub genes (IL7R, ITK, SOD1, CD3D, LEF1, FBL, MAF, DNMT1, and SLAMF1) may be associated with molecular subtypes and immune regulation of IS.

## 1 INTRODUCTION

Currently, cerebrovascular disease endangers human health and life, with high morbidity, disability and mortality [1]. Ischaemic stroke (IS), the primary type of the stroke, is a common cerebrovascular disease that occurs when the brain–blood circulation is interrupted by a clot blocking a blood vessel. Up to date, the diagnosis of IS depends on computed tomography and magnetic resonance imaging [2]. Magnetic resonance imaging is better than computed tomography for detecting IS within 6 h of stroke onset. However, the disadvantage of being expensive and less readily available limit magnetic resonance imaging as a diagnostic tool for IS. Therefore, it is desperately warranted to illustrate the underlying pathogenesis of IS and find novel biomarkers for IS.

Inflammation is related to arterial stiffening in healthy individuals [3]. In the progression of IS, inflammatory cell infiltration can activate a strong immune response and cause dysfunction of the immune microenvironment of central nervous system, which deteriorate the condition [4]. It has been reported that immune regulation can impede disease progression and ameliorate neurological function and prognosis, which highlights the significance of recovering the homoeostasis of the immune microenvironment of central nervous system [5, 6]. It is reported that immune‐inflammatory marker plasma levels are significantly associated with ischaemic lesion volume [7]. Thence, novel immunoregulatory therapeutic methods for IS are extremely urgent. Nevertheless, there are few systematic studies on the expression patterns of immune‐related gene modules in IS patients.

The rapid development of microarray and high‐throughput sequencing technology has provided numerous ways for the identification of biomarkers in dozens of diseases [8, 9, 10]. Weighted gene co‐expression network analysis (WGCNA) is a novel invented systematic biology tool that can be used to identify relevant patterns of the genes to obtain hub modules and hub genes for specific diseases [11, 12]. WGCNA addresses a shortcoming of traditional microarray analysis, which focuses only on individual genes and ignores the correlation between genes [13]. Through building gene co‐expression networks and recognition modules, WGCNA can study central genes closely related to clinical phenotypes, providing a new perspective for the finding novel molecular biomarkers and therapeutic targets in a variety of diseases [14, 15, 16]. It is noted that WGCNA has been used to construct co‐expression networks between genes and pathways to predict changes in related signalling pathways after IS [17]. In this study, identification of molecular subtypes of IS based on immune‐related genes (IRGs) and weighted co‐expression network analysis (WGCNA), may make contribution to discover potential targets for the treatment of IS, and laying a theoretical foundation for development of immunotherapy for IS.

## 2 MATERIALS AND METHODS

### 2.1 Data collection and preprocessing

Three gene expression datasets of IS (involving peripheral blood samples), GSE16561 (involving 39 cases and 24 normal controls), GSE22255 (involving 20 cases and 20 normal controls), and GSE58294 (involving 69 cases and 23 normal controls), were acquired from The Gene Expression Omnibus (GEO) database (http://www.ncbi.nlm.nih.gov/geo). For these three datasets, we downloaded the gene expression matrix file submitted by the author for analysis. The gene expression profile was annotated using the GPL6883 and GPL570 platform annotation files, and the gene probes were converted into gene symbols, in which the average value was taken for multiple probes correspond to the same gene. ComBat in the ‘sva’ R package was applied to the combined dataset to eliminate batch effects.

### 2.2 Identification of differentially expressed genes (DEGs) and collection of IRGs

The ‘limma’ package in R was utilised to screen DEGs between the IS and healthy controls. The screening standard of DEGs was set as false discovery rate (FDR) value < 0.05 and |log2FC| > 0.2. Volcano maps and heat maps were generated by the R package. A total of 1793 IRGs were downloaded from the ImmPort database (https://immport.niaid.nih.gov). Subsequently, the overlapped genes were obtained between DEGs and IRGs.

### 2.3 Consensus clustering analysis

On the basis of IRGs, 128 IS samples were clustered into *k* groups using the R package ConsensusClusterPlus (V1.48.0; maxK = 5, reps = 1000, pItem = 0.8, pFeature = 1, clusterAlg = ‘pam’). The optimal cluster number was confirmed by consensus matrix and the relative change in the area under the cumulative distribution function (CDF) curves. Then, two different IS groups (clusterA vs. clusterB) were obtained. DEGs between the two subtypes were screened using ‘limma’ package in R, and the screening criteria were set as FDR < 0.01 and |log2FC| > 0.3, and the volcano map was used for visualisation. Heat map was utilised to observe differential expression of IRGs.

### 2.4 Functional analysis

The function was analysed by Gene Ontology (GO) and Kyoto Encyclopaedia of Genes and Genomes (KEGG) pathway enrichment analysis. David was used for enrichment analysis of DEGs to study the biochemical processes and pathways that may involve the occurrence and development of IS. *P* < 0.05 terms was considered to be meaningful enrichment.

### 2.5 WGCNA

WGCNA is a typical systematic bioalgorithm to describe the correlation patterns between gene expression profiles and construct gene co‐expression networks. R package ‘WGCNA’ was used to analyse the co‐expression network of all DEGs, and a scale‐free gene co‐expression network was constructed. First, the ‘hclust’ function was used to cluster the sample data to detect outliers. Then, the ‘pickSoftThreshold’ function was used to select a suitable soft threshold power regulator to construct a scale‐free topology, and height set to 0.90. Subsequently, the adjacency matrix was calculated according to the kernel value, and the adjacency matrix was transformed into topological overlap matrix and corresponding dissimilarity matrix. Genes with similar expression patterns were clustered together and divided into modules by default parameters according to ‘cutree Dynamic’ function. Since the modules identified by the dynamic tree cutting algorithm may be similar, they were merged at a height of 0.25. Module eigengenes (ME) were defined as the first major component in each module, which can summarise the expression patterns of all genes in the module. In order to determine the most relevant key modules of IS, we used the ‘moduleEigengenes’ function to calculate the ME of each module, and used Pearson correlation coefficient analysis to analyse its correlation with IS. Finally, we selected the module with the highest correlation with IS as the hub module.

### 2.6 Identification of hub genes and protein–protein interactions (PPI) network establishment

We selected candidate hub genes according to the module connectivity and clinical traits of each gene in hub module. Module connectivity was defined as the absolute value of Pearson correlation between genes (Module Membership; MM). The relationship between clinical traits was defined as the absolute value (Gene Significance; GS) of Pearson correlation between genes and traits. Screening criteria of candidate hub genes was GS > 0.5 and MM > 0.7. In addition, we put all the genes in hub module into STRING database and imported them into Cytoscape (v 3.7.2) to construct PPI network. The top 55 genes were selected as the central nodes according to gene degree. We employed Venny 2.1 (https://bioinfogp.cnb.csic.es/tools/venny/index.html) to establish a Venn map of candidate hub genes in the WGCNA and the central genes in the PPI network, and the intersection genes were defined as real hub genes. The Pearson correlation between these hub genes was calculated by ‘cor’ function in R, and the expression of hub gene in the two subtypes and the control group was presented in a boxplot.

### 2.7 Receiver operating characteristic (ROC) curve analysis

The pROC, a package for R that contains a set of tools displaying, analysing, smoothing and comparing ROC curves in a user‐friendly, object‐oriented and flexible interface. The pROC package builds ROC curves and includes functions for computing confidence intervals, statistical tests for comparing total or partial area under the curve (AUC) or the operating points of different classifiers, and methods for smoothing ROC curves. In pROC, the ROC curves are empirical curves in the sensitivity and specificity space. AUCs are computed with trapezoids. In this study, we used the ROC curve to determine the accuracy of the real hub genes in distinguishing type A from type B in IS patients. The AUC represents the diagnostic value of real hub gene.

## 3 RESULTS

### 3.1 Identification of DEGs and collection of IRGs

**FIGURE 1:**
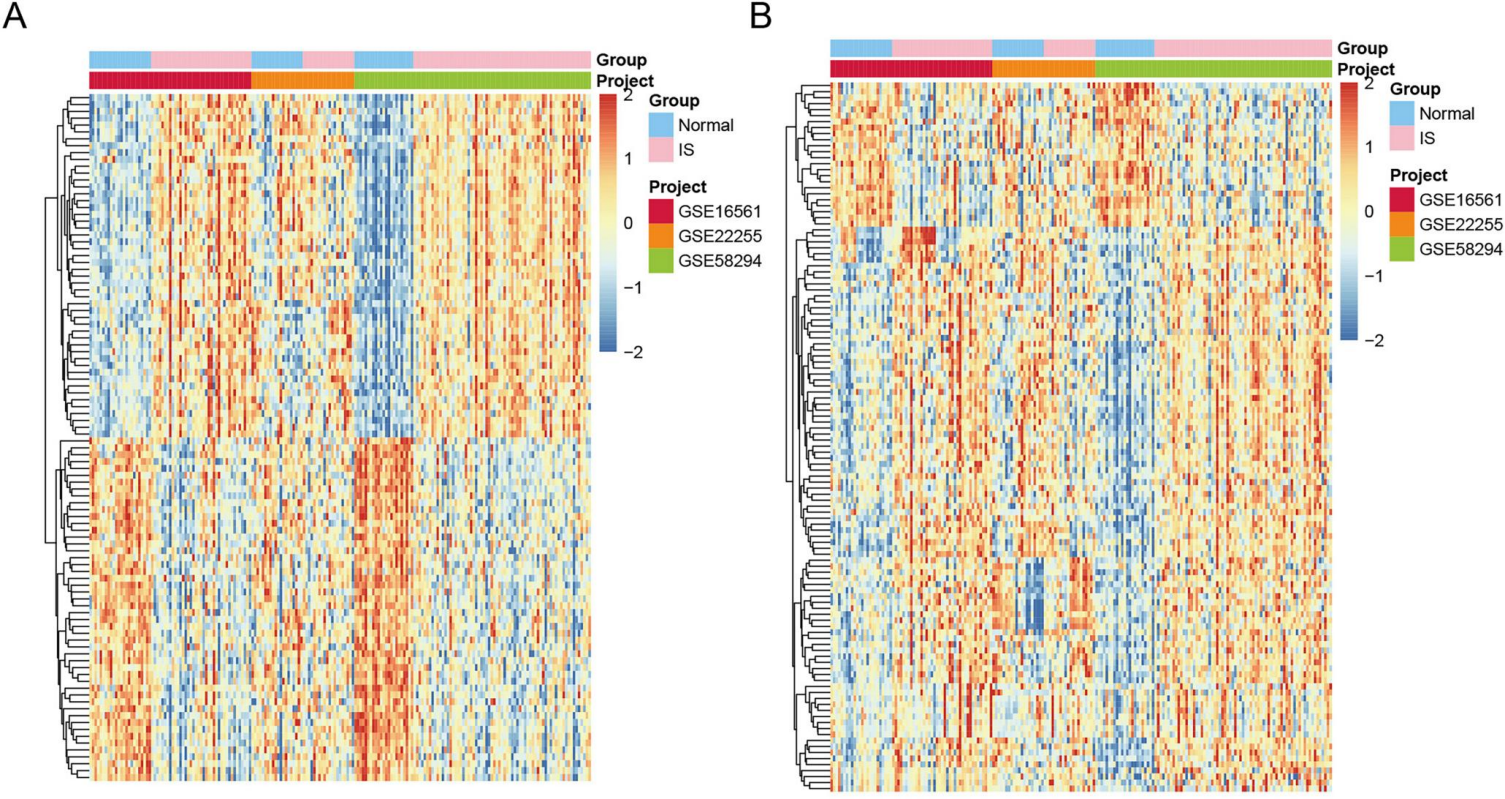
Heat map of top 100 differentially expressed genes (DEGs) and all immune‐related genes (IRGs) between ischaemic stroke (IS) and healthy controls. (a) Heat map of top 100 DEGs between IS and healthy controls. (b) Heat map of all IRGs between IS and healthy controls.

The details of GSE16561, GSE22255, and GSE58294 datasets are presented in Table 1. The combined datasets consisted of 67 healthy control samples and 128 IS samples. We found 827 DEGs that were differentially expressed between IS and healthy controls. Of which, 686 DEGs were up‐regulated and 141 DEGs were down‐regulated in IS compared to healthy controls. Heat map of top 100 DEGs is presented in Figure 1a. Subsequently, a total of 1793 IRGs were downloaded from ImmPort database, and the 1142 overlapped genes were obtained between DEGs and IRGs. A total of 118 differentially expressed IRGs were identified in IS compared to healthy controls, among which 94 IRGs were up‐regulated and 24 IRGs were down‐regulated. Heat map of these IRGs is shown in Figure 1b.

**TABLE 1:** Gene expression datasets used in this study.

GEO ID	Platforms	Control	IS	Source	Author
GSE16561	GPL6883	24	39	Peripheral blood	Barr TL
GSE22255	GPL570	20	20	Peripheral blood	Krug T
GSE58294	GPL570	23	69	Peripheral blood	Stamova
Total		67	128		

### 3.2 Consensus clustering analysis

**FIGURE 2:**
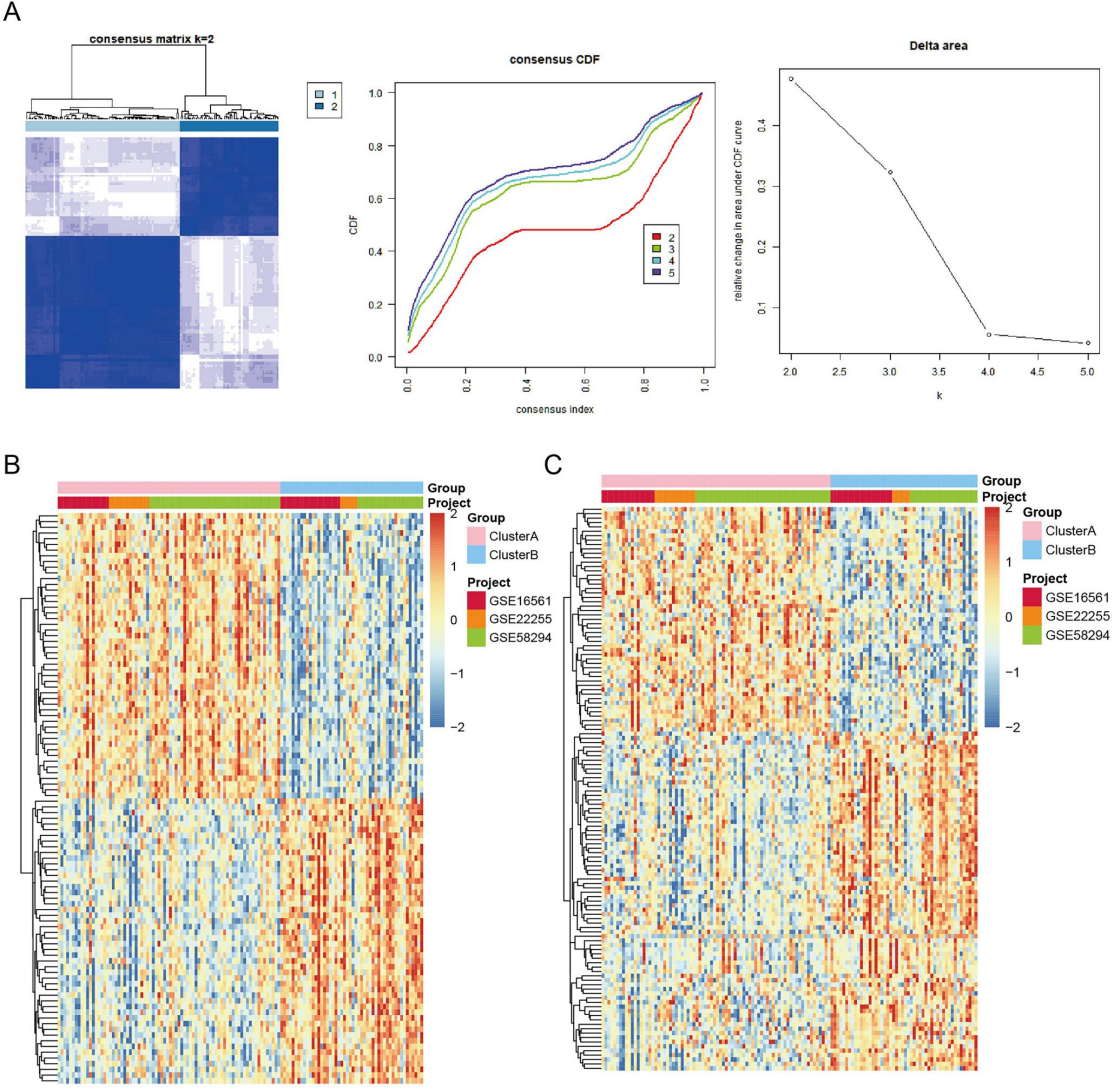
Consensus clustering identified two clusters of ischaemic stroke (IS) based on immune‐related genes (IRGs). (a) Under the consensus matrix for *k* = 2, 128 IS samples were clustered into two molecular subtypes: clusterA and clusterB. (b) Heat map of top 100 differentially expressed genes between clusterA and clusterB. (c) Heat map of all IRGs between clusterA and clusterB.

A total of 128 IS samples were clustered by R package ConsensusClusterPlus, and selected according to the consistency matrix *k* = 2 and CDF values (Figure 2a). In fact, 128 IS samples were also clustered into 3 (*n* = 53, 40 and 35), 4 (*n* = 50, 39, 34, and 5), 5 (*n* = 50, 40, 30, 5, and 3) molecular subtypes under the consensus matrix for *k* = 3, 4, 5 (Supplementary Figure S1). Consensus clustering revealed that the *k* = 2 was the most suitable choice to divide IS samples into two molecular subtypes with optimal clustering stability. Thence, 128 IS samples were clustered into two molecular subtypes: clusterA (*n* = 78) and clusterB (*n* = 50). Compared with clusterA group, 959 DEGs were identified in clusterB group, of which 626 DEGs were up‐regulated and 333 DEGs were down‐regulated. Heat map of top 100 DEGs between clusterB and clusterA is shown in Figure 2b. A total of 128 differential expressed IRGs in clusterB compared to clusterA, among which 77 IRGs were up‐regulated and 51 IRGs were down‐regulated. Heat map of these IRGs is shown in Figure 2c.

### 3.3 Functional analysis of DEGs between clusterB and clusterA

**FIGURE 3:**
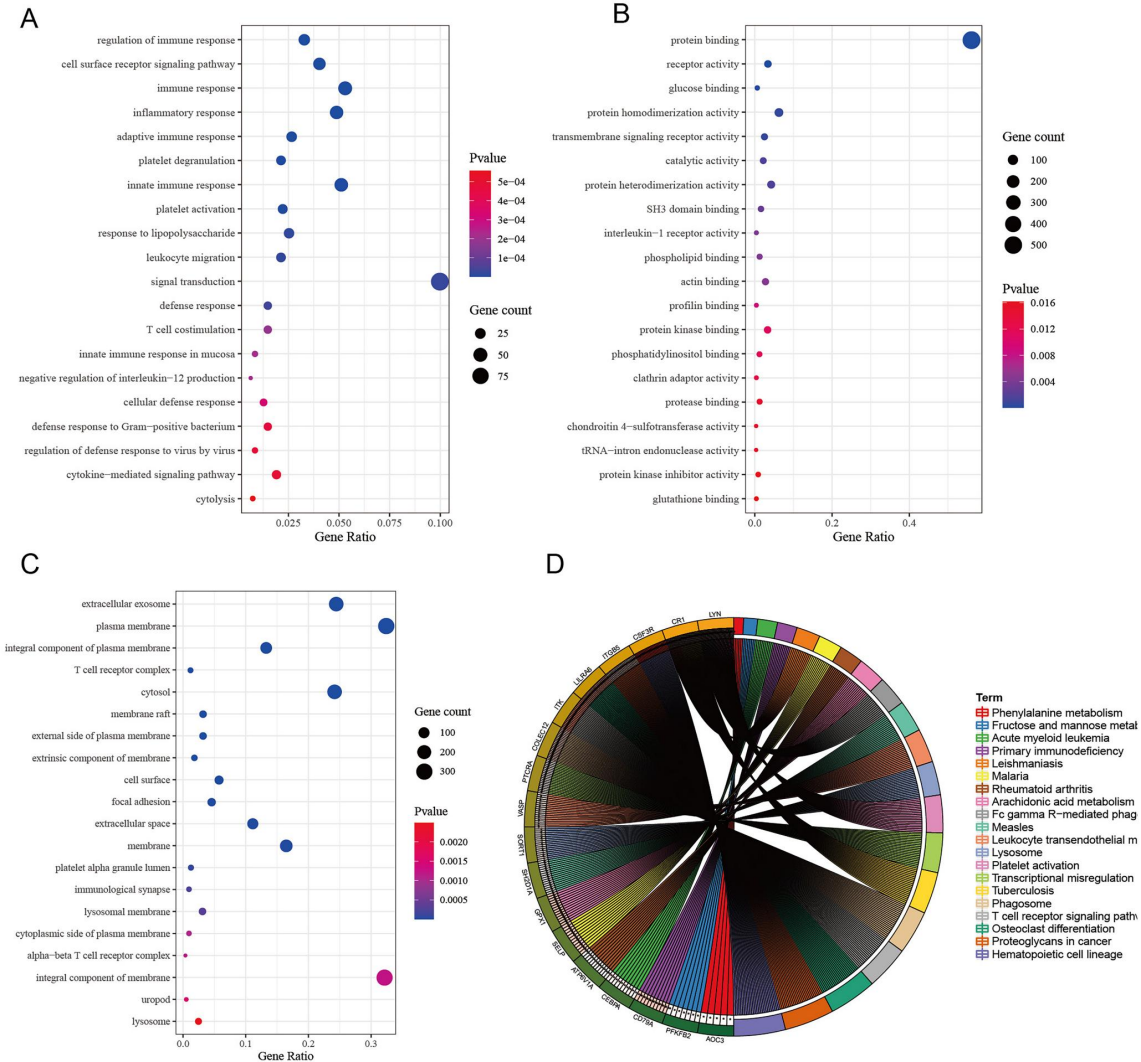
Functional analysis of differentially expressed genes (DEGs) between clusterB and clusterA. ((a)–(c)) The top 10 items of Gene Ontology analysis: biological processes, cellular components and molecular functions of the DEGs between clusterB and clusterA. (d) Kyoto Encyclopaedia of Genes and Genomes pathways analysis of the DEGs between clusterB and clusterA.

GO enrichment analysis found that these DEGs between clusterB and clusterA were mainly enriched in regulation of immune response, cell surface receptor signalling pathway, immune response, extracellular exosome, plasma membrane, integral component of plasma membrane, protein binding, receptor activity, and glucose binding (Figure 3a–c). The KEGG pathway enrichment analysis demonstrated that DEGs were primarily enriched in haematopoietic cell lineage, T cell receptor signalling pathway, osteoclast differentiation, arachidonic acid metabolism, primary immunodeficiency, phagosome, Fc gamma R‐mediated phagocytosis, and platelet activation (Figure 3d).

### 3.4 WGCNA construction and hub modules identification

**FIGURE 4:**
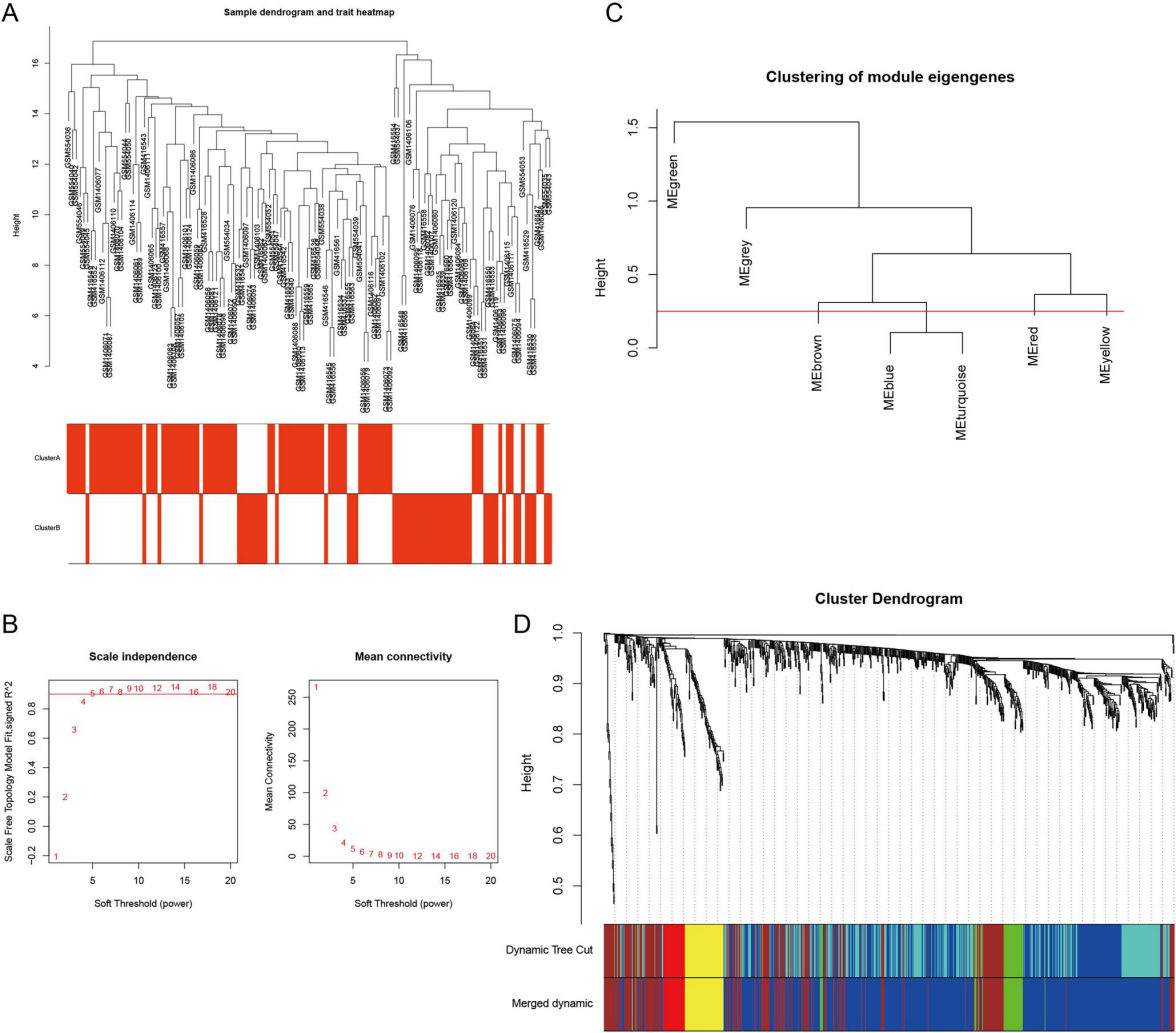
Weighted co‐expression network analysis reveals gene co‐expression networks. (a) The sample dendrogram and trait heatmap of 128 samples. (b) The power of *β* = 5 was selected as soft‐thresholding cut‐off standard power. (c) A total 5 distinct gene modules were identified. (d) The dendrogram of all genes is clustered based on a dissimilarity measure.

**FIGURE 5:**
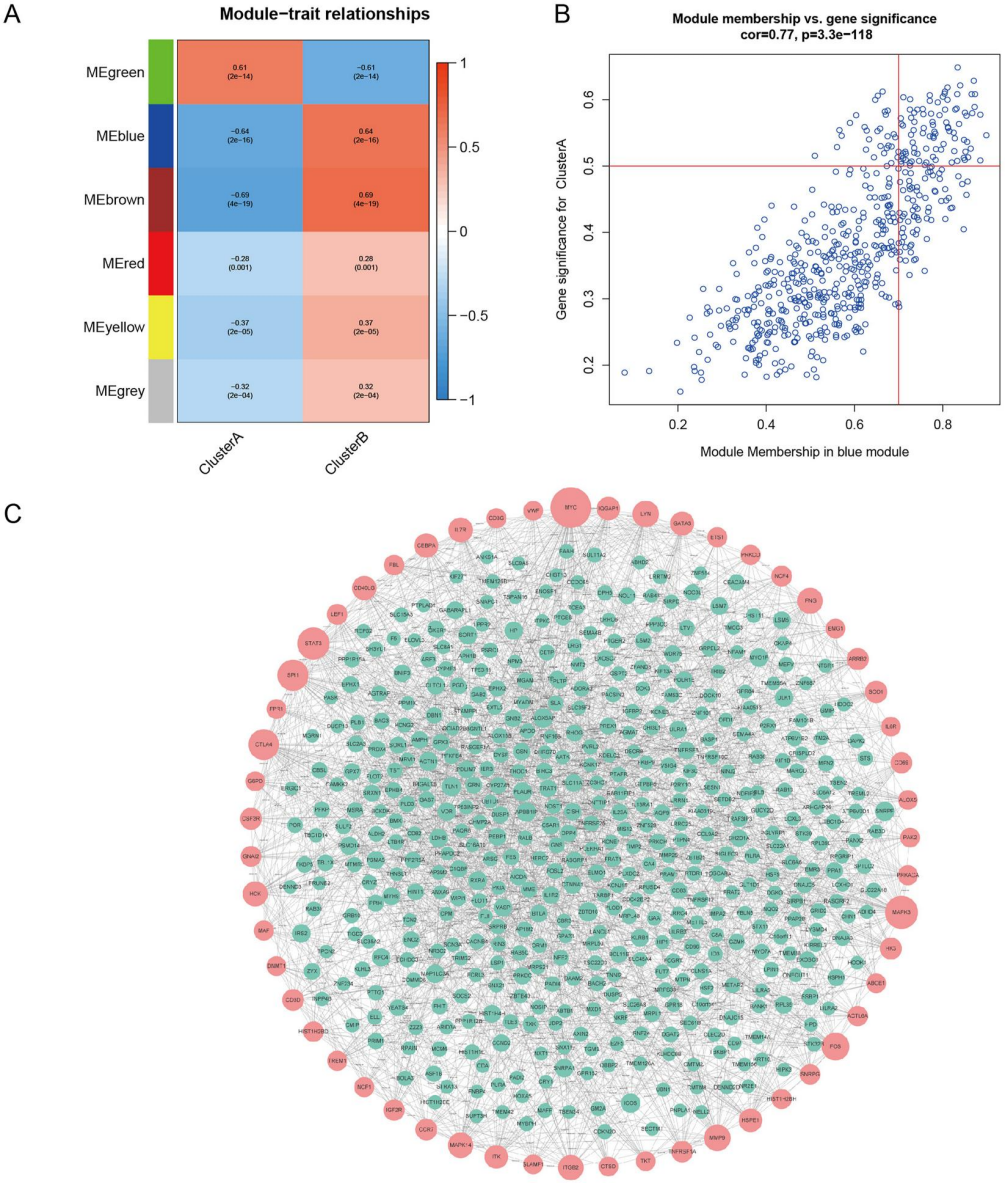
Identification of key modules and hub genes. (a) The heatmap shows the correlation between Module eigengenes and differentially expressed genes of ischaemic stroke (IS). (b) The scatter plot shows the correlation between gene significance for IS and module membership in blue module. (c) Protein‐protein interactions network of genes from the blue module.

WGCNA was executed to analyse 959 DEGs from 128 samples to identify genes associated with IS. The sample dendrogram and trait heatmap of 128 samples in our study were listed in Figure 4a. To build a scale‐free network, the power of *β* = 5 was selected as soft‐thresholding cut‐off standard power (Figure 4b). The dynamic tree cutting method was utilised to combine the modules with dissimilarity of <25%, and 5 modules were obtained (Figure 4c–d). Correlation analysis was executed between the eigengenes of each module and molecular subtypes of IS. Among the five modules, the blue module had the highest correlation with IS (*R* ^2^ = 0.64, *P* = 2e–16, Figure 5a), so the blue module was selected as the hub module, which contained a total of 597 genes. In the blue module, 90 genes were screened as candidate genes according to the criteria of GS > 0.5 and MM > 0.7, as shown in Figure 5b. A total of 597 genes in blue module was used to build PPI network, and this network contained 531 nodes and 1856 edges. The top 55 genes were selected as the central nodes according to gene degree (Figure 5c).

### 3.5 Real hub genes identification

**FIGURE 6:**
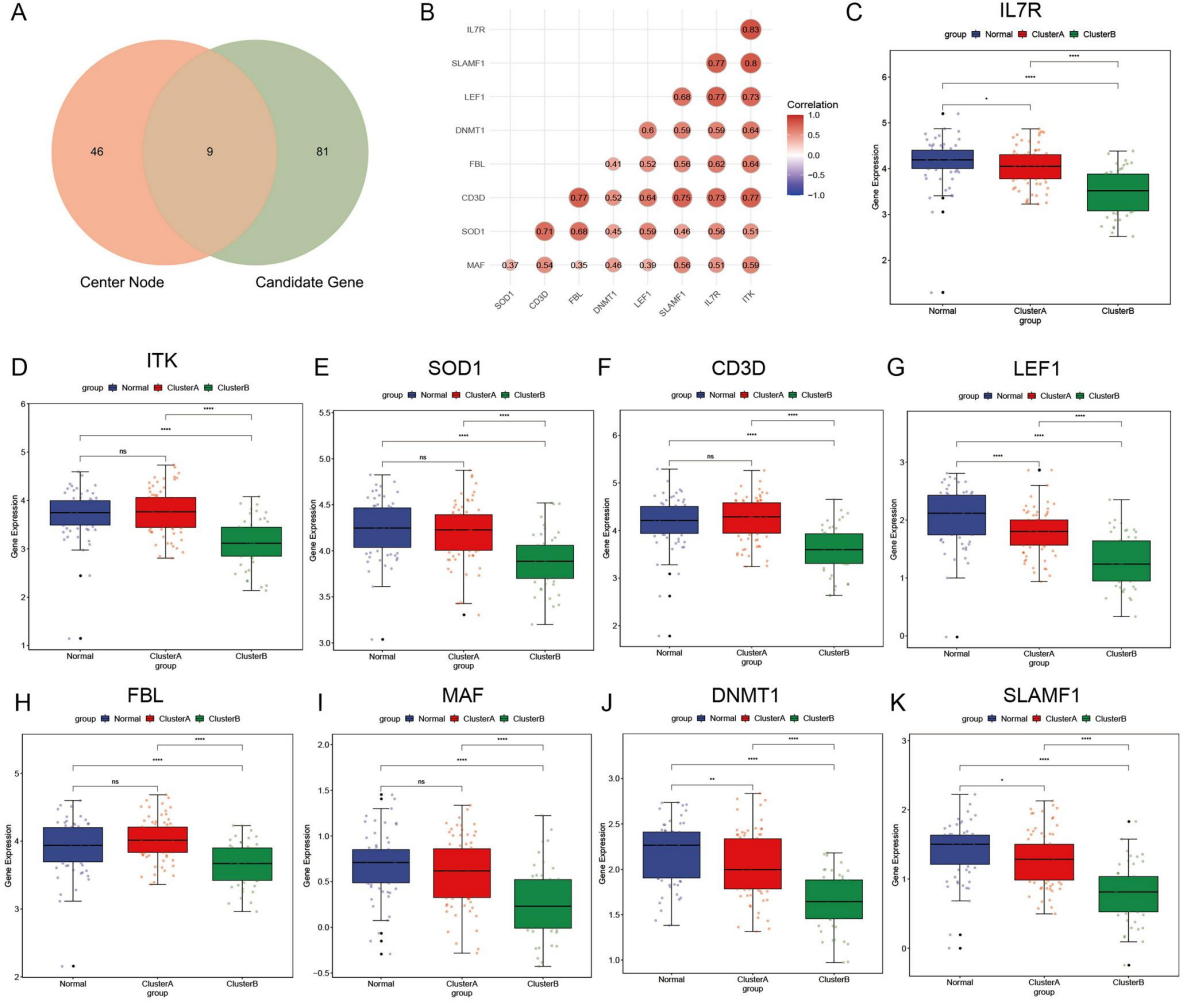
Identification of real hub genes. (a) Real hub genes were selected based on overlap between protein‐protein interactions and co‐expression networks. (b) Correlation analysis of nine genes. ((c)–(k)) The expression levels of nine real hub genes in ischaemic stroke.

The intersection of the screened candidate genes and the central node was taken, and nine genes were screened out, as shown in Figure 6a. We selected these nine genes as real hub genes, which were IL7R, ITK, SOD1, CD3D, LEF1, FBL, MAF, DNMT1, and SLAMF1. Then, we performed a correlation analysis of nine genes and found that there was a correlation between them (Figure 6b). In addition, we also showed the expression of the nine genes in the two subtypes and the normal group in the form of box plot. It was found that the nine genes were specifically high expressed in clusterA subtype and normal group, and low expressed in cluster B subtype (Figure 6c–k).

### 3.6 ROC curve analysis

**FIGURE 7:**
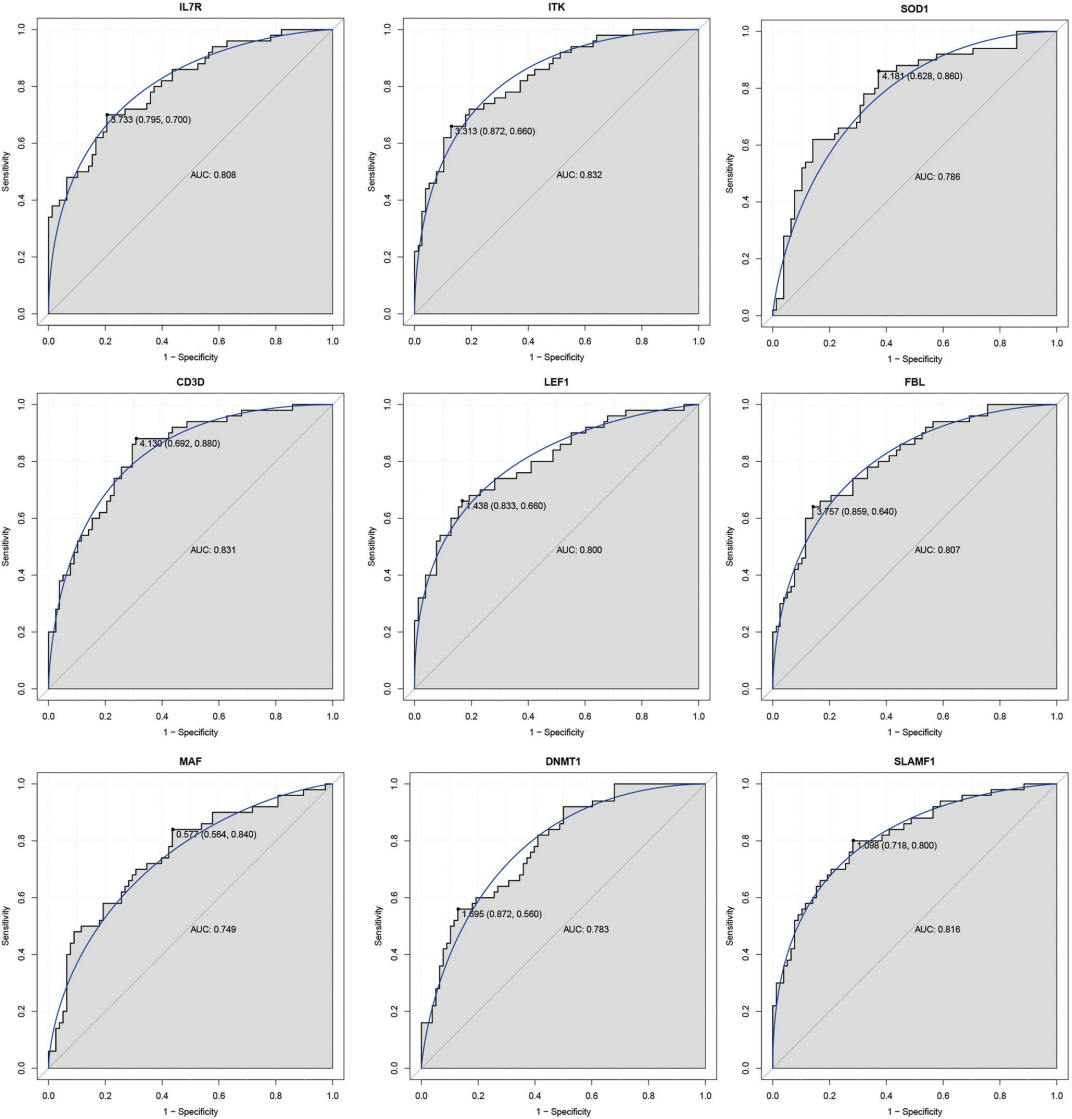
ROC analysis of nine real hub genes. The AUC was analysed to evaluate the performance of each hub genes. The *x*‐axis indicated 1‐specificity and *y*‐axis indicated sensitivity.

To further evaluate the accuracy of these nine real genes in distinguishing type A from type B in IS patients, ROC analysis was performed. The ROC analysis results showed that IL7R (AUC = 0.808), ITK (AUC = 0.832), SOD1 (AUC = 0.796), CD3D (AUC = 0.831), LEF1 (AUC = 0.800), FBL (AUC = 0.807), MAF (AUC = 0.749), DNMT1 (AUC = 0.783), and SLAMF1 (AUC = 0.816) were capable of discriminating in clusterB and clusterA of IS (Figure 7).

## 4 DISCUSSION

IS is one of leading cause of mortality and the primary cause of disability worldwide, placing a heavy burden on families and societies [18]. Previous reports have uncovered the genetic influences on inflammatory pathogenesis of IS [19, 20]. It is noted that studies have shown that IRGs play an important role in the development of IS [21]. Nevertheless, the IRGs involved in the progression of IS are largely unknown. Thus, it is extremely urgent to identify key IRGs and exploit new therapeutic targets for IS. In our study, we downloaded gene expression data of IS and healthy control samples from GEO database, and obtained 827 DEGs (686 up‐regulated genes and 141 down‐regulated genes) and 1142 IRGs between IS and healthy controls. On the basis of 1142 IRGs, 128 IS samples were clustered into two molecular subtypes: clusterA (*n* = 78) and clusterB (*n* = 50). Based on the WGCNA, we found the blue module had the highest correlation with IS. In the blue module, 90 genes were screened as candidate genes according to the criteria of GS > 0.5 and MM > 0.7. The top 55 genes were selected as the central nodes according to gene degree in PPI network of all genes in blue module. Through taking overlap, the nine real hub genes (IL7R, ITK, SOD1, CD3D, LEF1, FBL, MAF, DNMT1, and SLAMF1) were obtained, and these genes might distinguish between clusterA subtype and clusterB subtype of IS. As we know, in addition to IL7R, SOD1, DNMT1, and SLAMF1, the down‐regulated genes, including ITK, CD3D, LEF1, FBL, and MAF, in IS were reported for the first time in this study.

IL7R (interleukin‐7 receptor), a heterodimer composed by IL7R alpha chain and common gamma chain, is relatively common in the immune system, which plays a critical role in progression of human T‐cell [22]. Currently, the failure of Crohn's disease and ulcerative colitis treatment, has been reported to be closely related to the over expression of the IL‐7R signalling pathway, suggesting that IL‐7R is a fatal therapeutic target and potential biomarker for Crohn's disease and ulcerative colitis [23]. IL‐7R is found to be critical for development of T‐cell acute lymphoblastic leukaemia [22]. In childhood B‐cell precursor acute lymphoblastic leukaemia, IL7R has been reported to be associated with infiltration and relapse of central nervous system [24]. Moreover, the latest study discovers that IL7R is low expression in IS patients, which may be a new therapeutic target to promote functional recovery after IS [8]. In this study, IL7R was down‐regulated not only between IS group and healthy control group, but also between clusterB subtype and clusterA subtype of IS. The ROC analysis results found that IL7R (AUC = 0.808) was capable of discriminating in clusterB and clusterA of IS. Larger clinical samples and in‐depth studies are needed to uncover the function of IL7R in the progression of IS.

As an antioxidant enzyme, SOD1 (superoxide dismutase‐1) plays a key role in protecting cells from superoxide free radicals by maintaining the intracellular and extracellular oxidation/antioxidant balance [25]. The rsl041740 and rsl7880487 in the SOD1 gene have been found to be associated with cardiovascular death [26]. It has been found that rs7655372 of superoxide dismutase‐3 is associated with the risk of IS in the China Dali Region Han Population, and that the A and GA alleles of rs7655372 observably elevate the risk of IS [27]. However, the correlation between the SOD1 and the risk of IS is still unknown. Herein, we discovered that SOD1 was down‐regulated not only between IS group and healthy control group, but also between ClusterB subtype and ClusterA subtype of IS. And its role in IS require to be further clarified.

DNMT1 (DNA methyltransferase‐1) is a key enzyme involved in DNA methylation, which is needed to maintain DNA methylation patterns during cell replication [28]. The expression of DNMT1 in patients delayed cerebral ischaemia was higher than that in patients without delayed cerebral ischaemia [29]. DNMT1 modulates gene transcription via regulating allowable H3K4 and inhibitory H3K27 trimethylation in inhibitory intermediate neurons [30]. In HT22 cells, cytotoxicity induced by DNMT1 inhibitor 5‐Aza‐CdR is related to the changes in mRNA and protein expression of DNMT1, and decreased activity of DNMT1 may affect brain function [31]. MiR‐424 enhances H3K27me3 through the NFIA/DNMT1 signalling pathway to prevent astrocytes after cerebral ischaemia/reperfusion in elderly mice [32]. Tetrahydrocurcumin has been found to improve mitochondrial vascular dysfunction during IS by regulating DNMT1 [33]. In this study, we found that DNMT1 was down‐regulated in IS patients, indicating that DNMT1 may be a potential target for treatment of IS patients. And larger clinical samples and in‐depth studies are needed to expound the function of DNMT1 in the progression of IS.

SLAMF1 (signalling lymphocytic activation molecule family member 1) is an Ig‐like receptor that involves signal transduction networks in multiple immune cells [34]. Evidence has been suggested that SLAMF1 is involved in neutrophil autophagy during active tuberculosis [35]. SLAMF1 is found to regulate the autophagy of patients with chronic lymphocytic leukaemia and influence the response of chronic lymphocytic leukaemia cells to autophagy‐activated therapeutics [36]. SLAMF1 level is down‐regulated in chronic lymphocytic leukaemia and has an independent negative prognostic effect on overall survival in chronic lymphocytic leukaemia [37]. Recent studies indicate that SLAMF1 expression level is down‐regulated in IS patients, suggesting that SLAMF1 may be a potential therapeutic target for IS patients [8]. In the current study, we discovered that SLAMF1 was down‐regulated not only between IS group and healthy control group, but also between clusterB subtype and clusterA subtype of IS. However, the role of SLAMF1 in the immune regulation of IS has not been reported, and further research is necessary.

LEF1 (lymphoid enhancer factor‐1) is known for its important role in activating genes downstream of Wnt/β‐catenin. Recent literature has reported that LEF1 mediates the repressive effect of autophagic factor Beclin1 on induced cardiomyocytes formation [38]. ITK (IL2‐induced T cell kinase) has been shown to play an important role in T cell proliferation and differentiation. Selective targeting ITK has become an attractive method for the treatment of chronic intestinal inflammation and ulcerative colitis via driving the regression of mucosal inflammation [39]. CD3D is involved in encoding protein complexes, an important part of unique chains that bind TCR and ζ chains to form tCR‐CD3 complexes that promote T cell activation [40]. CD3D has been found to be related to several types of cancer, such as breast cancer [41], colon cancer [42], and bladder cancer [43]. Nevertheless, the function of LEF1, ITK, and CD3D in IS has not been found before. Here, this study revealed that LEF1, ITK, and CD3D were decreased in IS patients, suggesting that LEF1, ITK, and CD3D may be potential targets for treatment of IS patients. And larger clinical samples and in‐depth studies are required to illuminate the role of LEF1, ITK, and CD3D in the development of IS.

## 5 CONCLUSION

The real hub genes of IL7R, ITK, SOD1, CD3D, LEF1, FBL, MAF, DNMT1, and SLAMF1 may be associated with molecular subtypes and immune regulation of IS. This study may offer valuable theoretical basis for further elucidating the pathogenesis of IS and seeking new therapeutic targets. In addition, considering the high cost and limited availability for immediate access in CT and MRI‐based diagnoses, biomarkers for gene expression analysis‐based diagnosis may be suitable for addressing these challenges. Nevertheless, there were some limitations in this study. First of all, although some important hub genes related to IS has been screened, the potential mechanism has not been fully explored. Secondly, additional experimental analysis is needed to verify the results of our bioinformatics analysis. Thirdly, more clinical information (such as evaluation of IS subtypes according TOAST classification) needs to be collected and integrated into the following comprehensive analysis. Finally, the activity levels of the identified modules should be evaluated based on the gene expression of their member genes and see how these activity levels correlated with the diagnosis.

## AUTHOR CONTRIBUTIONS

Duncan Wei, Xiaopu Chen, Jing Xu, Wenzhen He. Conception and design: Wenzhen He; Administrative support: Wenzhen He; Provision of materials and samples: Duncan Wei, Xiaopu Chen and Jing Xu; Data collection and collation: Duncan Wei, Xiaopu Chen and Jing Xu; Data analysis and interpretation: Duncan Wei, Xiaopu Chen, Jing Xu, and Wenzhen He. All authors read and approve the publication of the article.

## CONFLICT OF INTEREST STATEMENT

The authors declare that they have no conflict of interest.

## Supporting information

Figure S1

## Data Availability

The datasets generated during and analysed during the current study are not publicly available, but are available from the corresponding author on reasonable request.
