# Supplementary material for: Identification of molecular subtypes of ischaemic stroke based on immune‐related genes and weighted co‐expression network analysis
Source: IET Syst Biol. 2023 Feb 18;17(2):58–69. doi: 10.1049/syb2.12059 (PMC10116020; doi:10.1049/syb2.12059)

**SUPPLEMENTARY FIGURE 1**

IS samples were clustered into 3, 4, 5 molecular subtypes under the consensus matrix for k = 3, 4, 5.


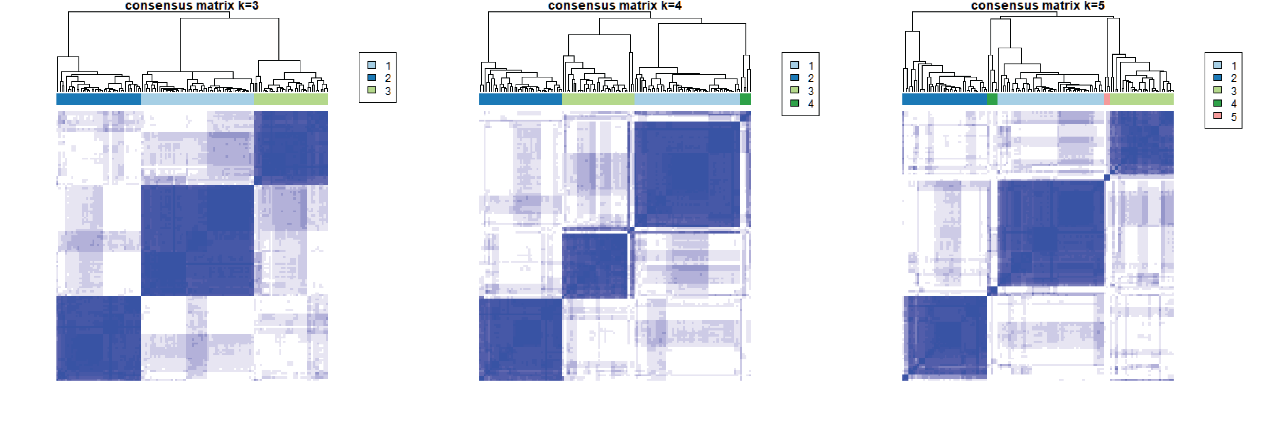

Supplement: Supplementary file 1 — Figure S1 [file SYB2-17-58-s001.docx]
